# Short-Term L-Citrulline Supplementation Does Not Affect Blood Pressure, Pulse Wave Reflection, or Arterial Stiffness at Rest and during Isometric Exercise in Older Males

**DOI:** 10.3390/sports11090177

**Published:** 2023-09-07

**Authors:** Andrea Tryfonos, Filippos Christodoulou, George M. Pamboris, Stephanos Christodoulides, Anastasios A. Theodorou

**Affiliations:** 1 Department of Life Sciences, School of Sciences, European University Cyprus, Nicosia 1516, Cyprus; a.tryfonos@euc.ac.cy (A.T.); f.christodoulou@external.euc.ac.cy (F.C.); g.pamboris@euc.ac.cy (G.M.P.); s.christodoulides@external.euc.ac.cy (S.C.); 2 Department of Laboratory Medicine, Karolinska Institutet, 171 77 Stockholm, Sweden; 3 School of Medicine, European University Cyprus, Nicosia 1516, Cyprus

**Keywords:** nitric oxide, dietary supplements, endothelium function, hypertension, cardiovascular disease

## Abstract

Hypertension and arterial stiffness are significant factors contributing to cardiovascular disease. L-citrulline, a nitric oxide precursor, has been proposed as a nutritional, non-pharmacological blood pressure-lowering intervention. This study aimed to investigate the impact of L-citrulline on central and peripheral blood pressure, pulse wave reflection, and central arterial stiffness at rest and during an isometric knee extension exercise protocol. Twelve older males received 6 g of L-citrulline or a placebo for six days using a double-blind crossover design. Blood hemodynamics parameters (i.e., aortic and brachial systolic and diastolic blood pressure, mean arterial pressure, pulse pressure, heart rate), pulse wave reflection (i.e., augmented pressure, augmentation index, forward/backward wave pressure), and arterial stiffness (i.e., carotid–femoral pulse wave velocity) were measured at baseline, post-supplementation, and during isometric exercise. No significant effects of L-citrulline supplementation were observed at rest or during exercise on blood pressure, pulse wave reflection, or arterial stiffness. Both central and peripheral blood pressure were increased during the exercise, which is consistent with isometric contractions. The results of the present study do not support any blood pressure-lowering effect of short-term L-citrulline at rest or during low-intensity isometric exercise compared to the pre-exercise values in older males.

## 1. Introduction

Cardiovascular disease is the number one cause of morbidity and mortality worldwide [1]. Several mechanisms have been identified as contributors to cardiovascular disease, including hypertension [2], endothelial dysfunction [3], and arterial stiffness [4]. Elevated blood pressure appears to trigger arterial stiffness, whereas increased arterial stiffness is associated with blood pressure progression [2,3,4], indicating a vicious cycle that contributes to an elevated risk of developing cardiovascular disease. Indeed, hypertension and arterial stiffness have been proposed as independent risk factors for cardiovascular conditions in the general population [5,6], further highlighting their clinical relevance.

Nitric oxide is a critical signaling molecule and a potent vasodilator that regulates vascular endothelial function [7,8]. In addition, nitric oxide is essential to skeletal muscle function [9] because it improves oxygen supply and metabolism [10] and redox status [11]. In the human body, nitric oxide is mainly generated through two main pathways: (i) endothelial nitric oxide synthases, which utilize oxygen and L-arginine to synthesize nitric oxide and L-citrulline, and (ii) the nitrate–nitrite–nitric oxide reduction pathway, which is stimulated during low oxygen availability or hypoxia [12]. Nitric oxide regulates vascular function by activating the soluble guanylate cyclase pathway in vascular smooth muscle cells [13], resulting in smooth muscle relaxation, widening of the blood vessel lumen, and improved blood flow [7,8].

Considering the pivotal role of nitric oxide in cardiovascular function, supplementation with nitric oxide precursors such as inorganic nitrates, L-arginine, and L-citrulline has been widely used to enhance nitric oxide bioavailability [14,15]. Supplementation with nitrates via a nitric oxide synthase-independent pathway appears to favorably affect blood pressure [16] and endothelial function [17]. In contrast, nitric oxide biosynthesis from L-arginine and L-citrulline is a nitric oxide synthase-dependent pathway that also appears to have favorable effects on cardiovascular health [18]. Regarding L-arginine and L-citrulline, L-arginine supplementation seemed to be the first option in this direction. However, it is highly degraded in the liver [19] and impairs nitric oxide synthesis [20]. Moreover, high levels of L-arginine may stimulate the enzyme arginase, leading to increased L-arginine catabolism [13]. In contrast, L-citrulline, a byproduct of nitric oxide synthesis via L-arginine oxidation, is efficiently recycled to L-arginine in the kidneys through the enzymes arginosuccinate synthase and arginosuccinate lyase, facilitating nitric oxide bioavailability [21]. Interestingly, oral supplementation of L-citrulline remains largely intact in the intestine and liver and is directly transported to the kidneys [22], resulting in higher plasma levels of L-arginine than direct supplementation with L-arginine [21]. Therefore, L-citrulline has been proposed as a potential non-pharmacological agent to enhance nitric oxide bioavailability, promoting cardiovascular health [18,23]. It is worth mentioning that pharmacokinetic studies have shown that short-term L-citrulline supplementation is safe and well tolerated [21].

L-citrulline has shown promising results as a blood pressure-lowering nutritional intervention, as it has been reported to reduce resting blood pressure [24,25,26,27]. These positive effects might be attributed to increased vasodilation as a result of higher plasma L-arginine concentrations and enhanced nitric oxide bioavailability [18,23]. Furthermore, a recent meta-analysis [28] demonstrated a more significant effect of L-citrulline supplementation on non-resting but transient arterial pressure changes induced by isometric exercise or cold exposure. This evidence is noteworthy, as arterial pressure elevation during exercise is often not controlled by antihypertensive medication, and it has been identified as an independent risk factor for cardiovascular events and mortality [29]. Exercise-induced increases in blood pressure can be caused by sympathetic and muscle metaboreflex-mediated vasoconstriction, an attempt of the body to increase blood flow to the working muscles [30]. However, the heterogeneity of the relevant studies (e.g., different supplementation doses, duration, and population under investigation) limits the ability to draw definitive conclusions regarding the effects of L-citrulline supplementation, and further research is required.

Even though several studies have examined the effect of oral L-citrulline supplementation on blood pressure and arterial stiffness, evidence has been limited to resting conditions or following cold pressure exposure. The present study investigated the impact of a six-day L-citrulline supplementation on aortic and brachial blood pressure and arterial stiffness at rest and during an acute isometric resistance exercise protocol in older males. Acute isometric knee extensor exercise was used to disturb hemodynamics, inducing significant increases in blood pressure [30]. We hypothesized that L-citrulline supplementation would have beneficial effects on blood pressure, pulse wave reflection, and arterial stiffness at rest. Additionally, during exercise, we expect that L-citrulline supplementation would attenuate the anticipated increases in blood pressure compared to the pre-exercise values in the L-citrulline group.

## 2. Materials and Methods

### 2.1. Participants

Twelve sedentary (<120 min/week of moderate-intensity exercise) healthy older males were recruited to participate in this study. Table 1 presents participant anthropometrics. Participants were informed of the purpose of the study and the methods used, and they provided written consent. The participants were included in this study if they were healthy and had had no history of musculoskeletal injury in the legs during the previous six months. They were instructed to refrain from vigorous physical activity for 48 h before the testing sessions. They were prohibited from drinking alcohol or caffeine within the last 24 h. Volunteers were non-smokers and received no medication or nutritional supplements in the previous three months before participating in the study. Researchers trained and motivated the participants to record their food intake for two days before the first session of the isometric exercise protocol. Participants were instructed to use that record and follow the same food intake before the second exercise session. These records were not provided to the researchers. In addition, participants were asked to maintain their usual lifestyle, including diet and physical activity, throughout the study.

### 2.2. Anthropometry

Prior to each trial, body mass was measured to the nearest 0.1 kg using an analogue balance scale (Seca 710, Hamburg, Germany), and height was recorded to the nearest 1 cm using a stadiometer (Seca 208, Hamburg, Germany). Using the seven skinfold method and the Siri equation [31,32], the body fat percentage was evaluated using a Harpenden Skinfold Caliper (John Bull, St. Albans, UK).

### 2.3. Experimental Design

**Figure 1:**
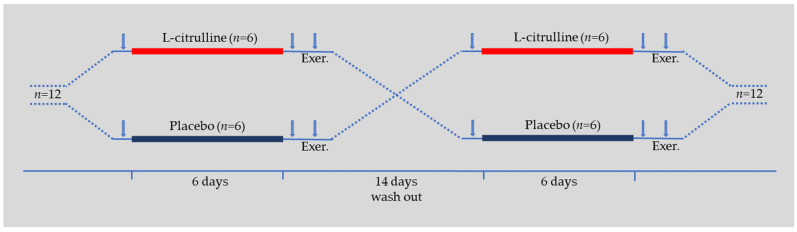
Experimental design. Arrows indicate the data collection time points (i.e., baseline, post-supplementation, and during isometric exercise). Exer, isometric exercise protocol.

This study was a randomized, double-masked, counterbalanced crossover placebo-controlled design. The experimental setup is shown in Figure 1. We provided a complete familiarization of the testing procedure before the data collection. Participants’ anthropometric characteristics and maximum voluntary isometric contraction (MVIC) of their right knee extensor muscles were measured on the first visit.

On the second visit, participants visited the laboratory to perform the first session of isometric exercise. Before the exercise session, baseline measurements were performed, including hemodynamic parameters, aortic pulse wave analysis, and arterial stiffness. Then, using a double-masked method, participants were randomly assigned to receive 6 g (3 g every 12 h) of L-citrulline between 08:00 and 10:00 in the morning and evening (Now, L-citrulline Pure Powder, Bloomingdale, IL) or a placebo (maltodextrin) daily for six days, starting from the next day after the visit (first treatment period). The group allocation of the participants was performed by an independent person using randomization software. The same independent person pre-packed L-citrulline or placebo in liquid form using identical bottles. Supplements were dissolved in 150 mL of water at 20 °C, and participants were instructed to wash the bottle immediately after consuming the supplement with 50 mL of water and drink it. Supplements were stored in the fridge (4 °C) immediately after production and discarded 24 h later. The independent researcher gave the participants the bottles daily, instructing them to keep them in the fridge. Each time, three bottles were provided to have an extra bottle in case of an accident. L-citrulline has a solubility of 200 mg/mL at 20 °C. The maximum amount that can be dissolved in 150 mL of water is 30 g. Therefore, the L-citrulline powder was fully dissolved. In addition, based on our previous study findings, this short-term supplementation can increase nitric oxide [30]. After completing the six days of supplementation, participants returned to the laboratory, and the same outcome measures were recorded. Testing sessions were performed within 12 to 14 h after the participants consumed the last dose of L-citrulline or placebo the night before to avoid the acute effects of the supplementation [20].

Then, the participants underwent a two-week wash-out period to avoid any potential carryover effects, after which the second treatment period started (placebo or L-citrulline) and followed the same procedures and measurements. The unblinding of the study occurred after the end of the statistical analysis.

### 2.4. Hemodynamics

After 10 min of rest in the seated position in a quiet room with the temperature set at 24 °C, blood hemodynamics were measured in duplicate using the SphygmoCor Xcel device (AtCor Medical, Sydney, Australia). Outcome measures included aortic and brachial systolic blood pressure (SBP), diastolic blood pressure (DBP), mean arterial pressure (MAP), aortic pulse pressure (PP), and heart rate (HR). Hemodynamic parameters were measured at baseline (rest), post-supplementation (rest, pre-exercise), and during the second minute of the isometric knee extension exercise. All measurements took place in the morning between 08:00 and 10:00 after an overnight fast.

### 2.5. Arterial Pressure Waveform Analysis and Pulse Wave Velocity

Sphygmocor Xcel was used to analyze the arterial pressure waveform. Briefly, with the participants seated, a conventional cuff oscillometer was tailored to the right arm centered over the brachial artery. The inflation began automatically through the device’s software for evaluating brachial systolic and diastolic pressure. Then the cuff deflated and inflated again below diastolic pressure to obtain a volumetric displacement signal representing the peripheral vascular waveform [33]. The parameters used in the study for the waveform analysis were augmented pressure (AP), augmentation index (AIx), augmentation index standardized at a heart rate of 75 beats per minute (AIx@75), and forward (Pf) and backward (Pb) wave pressure. Similarly, these parameters were measured at baseline before supplementation and post-supplementation at duplicates and during the second minute of the isometric exercise of the knee extensor muscles.

Carotid–femoral pulse wave velocity (cfPWV) was measured in duplicate in the supine position using the same equipment and method [33] to assess central arterial stiffness before and after the supplementation. The cfPWV was not measured during exercise. Briefly, the examiner fitted a femoral cuff to the upper side of the right thigh, and simultaneously, a tonometry was placed over the participant’s right carotid artery. The cfPWV was calculated as the transit time between the femoral and carotid pulse. Velocity measurement was calculated as the pulse wave difference in transit time between the upper side of the femoral cuff and the carotid artery, divided by the distance. The distance between the upper side of the femoral cuff and the carotid was measured with a validated measuring tape by subtracting the distance from the carotid to the suprasternal notch. cfPWV was measured pre- and post-supplementation.

### 2.6. Isometric Knee Extensor Exercise

During the participant’s first visit, their MVIC of the right leg knee extensor muscles was measured using an electronic dynamometer (K-Link, Kinvent, Montpelier, France), which is a dynamometer used in the evaluation and rehabilitation of muscle strength that provides real-time feedback [34,35].

Participants were seated on an adjustable chair with their legs dangling and their knees bent to 90° [35]. A strap attached to a solid bar was connected to the device and fitted around the participants’ right ankle. Participants were then asked to push forward (straighten their knee) as hard as possible for five seconds without moving their trunks, followed by 60 s of rest between repetitions. Three trials were recorded, and the data were stored using the KFORCE APP (Kinvent, Montpelier, France). The average of the two closest values was calculated as the MVIC.

After assessing the knee extensor MVIC, the researchers conducted the isometric exercise protocol based on each participant’s MVIC. The exercise protocol consisted of two minutes of isometric exercise of the right knee extensor muscles at 30% of their MVIC. The setup was the same as the one used during the evaluation of the MVIC. Visual feedback was provided to the participants through a tablet screen during the exercise to ensure that the desired force was achieved [36]. After the end of the test, participants remained seated for three minutes for recovery.

### 2.7. Statistical Analysis

Data are presented as means and standard deviations (SD). Normal data distribution was tested and confirmed using the Shapiro–Wilk test for all dependent variables. A two-way repeated-measures ANOVA test ((group (L-citrulline vs. placebo) × time (baseline, post supplementation, and during the second minute of isometric exercise)) was performed for aortic and brachial SBP, DBP, MAP, HR, AP, AIx, Pf, and Pb. A second two-way repeated-measures ANOVA test was performed for cfPWV ((group (L-citrulline vs. placebo) × time (baseline and post-supplementation)). If a significant interaction was found, pairwise comparisons were performed using the Sidak test. Statistical significance was accepted a priori at *p* < 0.05. Data were analyzed using the SPSS 25 statistical package (SPSS Inc., Chicago, IL, USA).

An a priori power analysis was performed (G*Power v. 3.1.9.7, Heinrich Heine University, Düsseldorf, Germany) to calculate the minimum number of participants in the study [37]. For an F test (repeated measures, within–between interaction factors for three time points), the minimum total number of participants for a statistical power of 0.80, an effect size of 0.27, and a significance level of 0.05 required a total sample size of 24. Therefore, 12 volunteers were recruited, as the study had a counterbalanced crossover design.

## 3. Results

### 3.1. Hemodynamics

**Figure 2:**
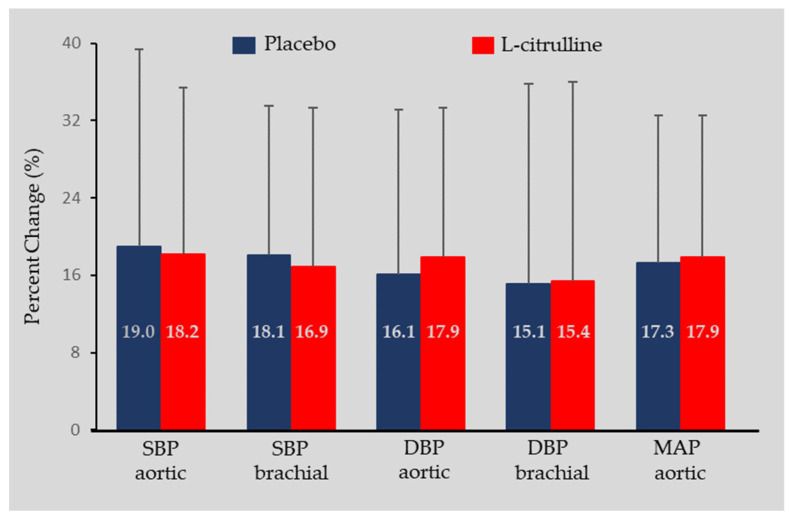
Blood hemodynamic percent changes after supplementation during isometric exercise protocol compared to pre-exercise values (mean ± SD).

No statistically significant condition by time interaction or main effect of the condition was found for aortic or brachial SBP, DBP, MAP, or HR. However, a significant main effect of time was found for all parameters (Table 2). Specifically, both conditions exhibited similar increases during isometric exercise compared to post-supplementation for aortic SBP (L-citrulline condition: +20.8 mmHg, 95% CI [5.1, 36.4] and placebo condition: +21.3 mmHg; 95% CI [5.6, 37.0]) and for aortic DBP (L-citrulline condition: +14.4 mmHg, 95% CI [4.4, 24.3] and placebo condition: +13.4 mmHg, 95% CI [3.4, 23.3]. Similar increases for both conditions were also found for brachial SBP and DBP (L-citrulline condition: +20.0 mmHg, 95% CI [5.6, 34.3] and placebo condition: +23.9 mmHg, 95% CI [9.5, 38.2]) and (L-citrulline condition: +12.5 mmHg, 95% CI [−0.2, 25.0] and placebo condition: +13.1 mmHg, 95% CI [0.6, 25.7]), respectively. For MAP, similar increases were observed for both conditions (L-citrulline condition: +17.0 mmHg, 95% CI [7.2, 26.3] and placebo condition: 16.6 mmHg, 95% CI [7.2, 26.3]). For HR, a significant main effect of time was found, with similar increases in both conditions (L-citrulline condition: +14.4 bpm, 95% CI [8.5, 20.3] and placebo condition: +12.7 bpm, 95% CI [6.8, 18.6]). No significant main effect of time, condition, or condition-by-time interaction was found for aortic PP. The percentage changes in blood hemodynamic parameters during exercise compared to the pre-exercise values are presented in Figure 2.

### 3.2. Arterial Pressure Waveform Analysis and Pulse Wave Velocity

No statistically significant main effect of time, condition, or condition-by-time interaction was found for AP, AIx, AIx@75, Pf, Pb, or cfPWV (Table 3).

## 4. Discussion

The primary aim of this study was to examine the effect of L-citrulline supplementation for six days on central and peripheral blood pressure at rest and during blood pressure attenuations induced by isometric exercise in healthy older males. Additionally, arterial stiffness was assessed at rest, and pulse waveform analysis was performed at rest and during isometric exercise. In contrast to our hypothesis, our findings indicate that six days of oral L-citrulline supplementation did not affect resting aortic or brachial blood pressure, arterial stiffness, or pulse wave reflection in older males. In addition, no effects on blood pressure or pulse wave reflection were observed after the supplementation during the second minute of the low-intensity isometric exercise test compared to the pre-exercise values.

In the present study, older males were recruited. It is worth noting the higher prevalence of increased blood pressure [38,39], endothelial dysfunction, and impaired nitric oxide synthesis [36] in this age group. Therefore, supplementation with L-citrulline was targeted, as it has been suggested to have favorable effects on blood pressure [27] and endothelial dysfunction [24]. The isometric exercise was chosen as a physiological stimulus to induce blood pressure elevation and investigate whether a non-pharmacological intervention (i.e., L-citrulline) can affect blood pressure during this activity compared to pre-exercise values. It is worth mentioning that low-intensity isometric muscle contractions are involved in everyday activities, such as carrying shopping bags. Thus, any blood pressure attenuation from L-citrulline supplementation at rest or during exercise would have substantial clinical application.

### 4.1. L-Citrulline and Blood Pressure

Increased blood pressure is considered a significant risk factor for cardiovascular disease progression, and interventions leading to lowering blood pressure are considered an effective strategy to avoid adverse effects on cardiovascular health. More specifically, it has been suggested that non-pharmacological nutritional factors, such as L-citrulline, could positively impact vascular health [18]. As a nitric oxide precursor, L-citrulline supplementation increases nitric oxide synthesis and bioavailability [40,41,42]. Nitric oxide plays a crucial role in regulating vascular endothelial function and blood pressure by relaxing the smooth muscle cells and expanding the vascular lumen [7,8]. However, it is essential to note that although the evidence regarding the L-citrulline effect on blood pressure is encouraging [18,43], the number of investigations is limited. Further studies are required to confirm the potential of L-citrulline supplementation in reducing blood pressure.

The present study found that a six-day supplementation does not affect resting blood pressure (aortic or brachial). These findings are consistent with similar studies that have investigated the effects of L-citrulline supplementation on resting blood pressure in young and older adults using the same dosage, ranging from seven days to four weeks, and found no effect of L-citrulline on resting blood pressure [40,44,45,46]. However, opposing results have been reported in other studies. A significant decrease in resting systolic blood pressure was found in a combined group of young (n = 7) and older men (n = 4), with elevated blood pressure after supplementation with 6 g of L-citrulline for seven days [47]. In this study, there was a significant decrease within the L-citrulline group, with no difference between the L-citrulline group and the placebo group. In a different study, it was reported that a four-week supplementation of 10 g of L-citrulline significantly lowered resting diastolic aortic blood pressure and MAP in hypertensive postmenopausal women [24]. Additionally, favorable effects on arterial systolic blood pressure supplementation were reported in young males during exposure to cold in an environmental chamber after 14 days of L-citrulline [25]. Furthermore, reductions in brachial and aortic systolic and diastolic blood pressure were found in postmenopausal obese women [26,27] after 4 and 8 weeks of L-citrulline supplementation. It is worth noting that three of the studies that reported decreases in blood pressure recruited hypertensive postmenopausal obese individuals [24,26,27]. In the present study, older yet healthy males were recruited, as their blood pressure values are considered pre-hypertensive and without clinical cardiovascular diseases that would justify pharmacological prescription. Thus, the participants’ clinical condition in these studies [24,26,27] might have influenced the response to L-citrulline supplementation, as obesity and menopause are associated with hypertension progression and development [48]. Furthermore, except for one study that followed short-term supplementation [47], the rest followed a more prolonged supplementation, ranging from four to eight weeks, which could have influenced the effectiveness of the intervention. Nevertheless, it would be interesting for future studies to compare the impact of short-term versus more extended supplementation protocols on young and older individuals with normal and high blood pressure levels.

Besides resting blood pressure, this study was the first to assess the effect of short-term use of L-citrulline supplementation on exercise-induced blood pressure increases, specifically during acute isometric knee extensor exercise. We have shown that two minutes of isometric knee extensor exercise at 30% of pre-determined maximum strength is adequate to elevate both peripheral and central blood pressure significantly. To the best of our knowledge, only one study has assessed the impact of L-citrulline on exercise-induced blood pressure increase without any other intervention (e.g., cold exposure), using isometric handgrip exercise as a stimulus [49]. In that study, the authors reported lower aortic and brachial SBP and lower aortic DBP during exercise in the group that received 6 g of L-citrulline for two weeks. Thus, a more extended supplementation may be required to attenuate blood hemodynamic increases during isometric exercise. Furthermore, participants in that study were obese, which might have enhanced the blood pressure-lowering effects of L-citrulline, as decreases were also observed in obese postmenopausal women at rest [24,26,27]. Obesity is a chronic medical condition that negatively affects vascular health [50]. Considering the observations above, it is likely that L-citrulline supplementation may present a more significant effect on blood pressure in subjects with prior endothelial dysfunction, such as obesity, or that L-citrulline benefits might be sex-specific.

Given that exercise-induced blood pressure augmentation has been proposed as a better predictor of the development of cardiovascular disease in middle-aged individuals [51], we emphasize the need for future studies assessing the efficiency of supplements on hemodynamics to not only focus on resting conditions but also evaluate transient changes in response to exercise.

### 4.2. L-Citrulline and Arterial Stiffness

In addition to blood pressure, L-citrulline appears to favor arterial stiffness, assessed through measurements of pulse wave velocity at rest or following a combined cold pressure test with isometric exercise [40,44,49]. Conversely, in our study, six days of oral L-citrulline supplementation did not improve arterial stiffness in older men, as indicated by measurements of cf-PWV. These observations contrast previous research that reported reduced resting PWV [49]. Occhai et al. [40] and Figueroa et al. [49] reported decreases in brachial ankle PWV (baPWV) after one and two weeks of L-citrulline, respectively. In addition, Figueroa et al. [49] found reduced baPWV and femoral ankle PWV (faPWV) after eight weeks of L-citrulline, whereas no changes were observed in cfPWV. Therefore, the different arterial segments in which PWV was measured might explain the differences between those studies and our investigation. Subsequently, as baPWV mainly indicates peripheral arterial stiffness and cfPWV indicates central arterial stiffness [50], L-citrulline might be effective in decreasing peripheral but not central arterial stiffness. Nevertheless, other studies failed to observe any resting cfPWV changes within the same time frame [46,52], which somehow brings into question the effect of short-term L-citrulline supplementation on central arterial stiffness. Notably, two of the studies showing improvements in resting baPWV [40,44] after L-citrulline supplementation recruited individuals with baPWV values > 14 m/s, which is the cutoff value for the risk prediction of developing cardiovascular disease [53]. Our study’s baseline values for cfPWV were 7.5 m/s; thus, L-citrulline may be efficient only in individuals with increased PWV. This evidence supports the finding that individuals at a higher risk for cardiovascular disease development experience more pronounced hemodynamic changes following L-citrulline supplementation than healthy individuals [54].

### 4.3. L-Citrulline and Aortic Pulse Wave Reflection

Furthermore, we assessed the effect of L-citrulline supplementation on aortic pulse wave reflection at rest after six days of supplementation and after supplementation during isometric exercise as a physiological stimulus of hemodynamic disturbances. Even though leg isometric exercise increased AIx, AIx@75, and backward wave pressure compared to the pre-exercise values after the supplementation, these changes were not statistically significant in our study. It is worth noting that, in the present study, the responses to exercise were not evaluated before and after supplementation but only after supplementation. Previous studies that evaluated the response from rest to exercise using isometric handgrip exercise in young men and women reported significant increases in aortic pulse wave reflection [55,56].

Nevertheless, studies with L-citrulline supplementation have reported varying results regarding the impact on AIx at rest, during the cold pressor test, or during concurrent cold pressor and isometric handgrip tests [25,49]. The only study that evaluated the impact on AIx after two weeks of 6 g of L-citrulline during only the handgrip isometric exercise test reported a reduction of AIx in overweight men [49]. Therefore, further studies are needed to clarify the effects of L-citrulline on AIx responses to isometric exercise. Assessing the impact of supplements, drugs, and other interventions in response to isometric exercise increases the clinical relevance of the studies. These responses may better reflect the real-world situations where cardiac events can be triggered in asymptomatic older individuals during everyday activities.

### 4.4. Limitations

The lack of any measurement of nitric oxide production, which could have provided us with valuable mechanistic insights into nitric oxide metabolism, is a limitation of the present study. We suggest future nutritional interventions incorporating nitric oxide measurements in various compartments, such as plasma and muscle, when evaluating the effects of dietary interventions with nitric oxide precursors. Additionally, it is crucial to recognize that blood pressure regulation and the development of cardiovascular diseases can vary between males and females throughout [57,58]. Although there is evidence suggesting that differences between men and women in regards to endothelial function and cardiovascular diseases following menopause may become less obvious, as women have no longer protection from estrogen [59], recruiting males only in our investigation limits our findings’ generalizability to females. Thus, the exclusion of women is a major limitation of the present study. Yet, the fact that in the present study, L-citrulline was given in a fluid form rather than having participants consume capsules differs our study supplementation methodology from similar studies that mainly provided L-citrulline in capsules. Furthermore, our supplementation duration was only six days, whereas a more extended supplementation might be more effective.

Moreover, a limitation of the present study is that for the sample size calculation, we used a standard small to medium effect size and not the effect size from previous experiment’s data. Based on the observed effect sizes in the study, the value used for the sample size calculation was high. Another major limitation is the evaluation of blood hemodynamics responses to exercise only after supplementation and not comparing with responses before supplementation.

### 4.5. Conclusions

Our results did not find evidence to support any blood pressure-lowering effect of 6 g of L-citrulline supplementation for six days or any favorable impact on pulse wave reflections or arterial stiffness in older males at rest or during isometric exercise, which causes significant elevations in blood pressure. Although it has been suggested that L-citrulline may reduce blood pressure [18,28,43], we believe that more research is necessary to support this potential role, as the number of studies is limited and the findings are inconsistent. Most studies reporting decreases in blood pressure after L-citrulline supplementation were observed during disturbed hemodynamics (e.g., during the cold pressor test with or without concurrent exercise), whereas findings for resting central and peripheral blood pressure are not solid. Yet, the short-term supplementation period followed in our investigation may not suffice to induce these beneficial changes in blood pressure, pulse wave reflection, and arterial stiffness, as demonstrated in other L-citrulline studies with longer supplementation at rest or during disturbed hemodynamics [25,44,46,49,52]. Furthermore, a recent study by Figueroa et al. [60] observed improvements in endothelial function in healthy postmenopausal women following four weeks of combined 2 g of citrulline with glutathione supplementation. In contrast, no changes were reported following 6 g of L-citrulline solely. As such, perhaps combining L-citrulline with other antioxidant compounds may be a more promising strategy to improve vascular health.

It remains of particular interest to select older yet healthy persons for this study who could benefit from L-citrulline supplementation, as they are generally characterized by lower endothelial function and pre-hypertension but have not yet developed clinical cardiovascular diseases to justify pharmacological prescription.

## Figures and Tables

**Table 1:** Participants’ anthropometric characteristics (mean ± SD).

	*n* = 12
Age (y)	64.3 ± 5.1
Height (cm)	173.9 ± 4.2
Weight (kg)	78.4 ± 6.9
BMI (kg/m^2^)	26.0 ± 2.7
Body fat (%)	26.8 ± 2.6
Waist circumference (cm)	100 ± 5.6
Hip circumference (cm)	105.8 ± 6.8
Waist-to-hip ratio	0.95 ± 0.02

**Table 2:** Aortic and brachial blood hemodynamics at baseline, post-supplementation, and during the isometric exercise protocol (mean ± SD).

		Post Supplementation	During Exercise	Interactions and Main Effects			Partial Eta-Squared		
	Baseline			C × T	C	T	C × T	C	T
Heart rate (bpm)									
Placebo	64.1 ± 6.3	64.0 ± 7.9	76.8 ± 11.5	*p* = 0.631	*p* = 0.963	*p* < 0.001	0.004	0.001	0.126
L-citrulline	63.5 ± 5.9	63.7 ± 6.1	78.1 ± 9.0						
Aortic SBP (mmHg)									
Placebo	121.9 ± 13.3	121.2 ± 12.7	142.5 ± 17.4	*p* = 0.899	*p* = 0.822	*p* < 0.001	0.002	0.002	0.538
L-citrulline	120.0 ± 11.5	121.1 ± 11.3	141.8 ± 14.4						
Brachial SBP (mmHg)									
Placebo	130.8 ± 13.2	129.4 ± 12.6	152.5 ± 21.6	*p* = 0.553	*p* = 0.788	*p* < 0.001	0.001	0.000	0.563
L-citrulline	131.5 ± 12.6	130.1 ± 14.5	151.8 ± 23.8						
Aortic DBP (mmHg)									
Placebo	83.3 ± 6.1	84.1 ± 6.5	97.5 ± 14.8	*p* = 0.919	*p* = 0.608	*p* < 0.001	0.001	0.012	0.527
L-citrulline	82.1 ± 5.9	81.9 ± 7.2	96.3 ± 9.0						
Brachial DBP (mmHg)									
Placebo	83.4 ± 6.0	84.2 ± 7.9	97.3 ± 21.5	*p* = 0.808	*p* = 0.652	*p* < 0.001	0.003	0.009	0.379
L-citrulline	82.8 ± 7.6	82.1 ± 6.5	94.6 ± 17.1						
Aortic MAP (mmHg)									
Placebo	99.2 ± 8.0	99.4 ± 8.2	116.0 ± 13.0	*p* = 0.962	*p* = 0.660	*p* < 0.001	0.001	0.009	0.618
L-citrulline	97.7 ± 6.6	98.1 ± 7.2	115.1 ± 12.6						
Aortic PP (mmHg)									
Placebo	38.6 ± 11.4	37.1 ± 10.7	45.01 ± 18.7	*p* = 0.856	*p* = 0.876	*p* = 0.071	0.004	0.001	0.126
L-citrulline	37.9 ± 11.5	39.2 ± 11.4	45.5 ± 12.0						

SBP, systolic blood pressure; DBP, diastolic blood pressure; MAP, mean arterial pressure; PP, pulse pressure; bpm, beats per minute; mmHg, millimeter of mercury; C × T, two-way interaction between condition and time; C, main effect of condition; T, main effect of time.

**Table 3:** Pulse wave analysis and arterial stiffness at baseline, post-supplementation, and during the isometric exercise protocol (mean ± SD).

		Post Supplementation	During Exercise	Interactions and Main Effects		
	Baseline			C × T	C	T
Augmented pressure (mmHg)						
Placebo	10.8 ± 4.4	11.0 ± 4.4	11.3 ± 5.7	*p* = 0.537	*p* = 0.730	*p* = 0.286
L-citrulline	11.1 ± 4.4	11.2 ± 4.0	12.8 ± 6.8			
Augmentation index (%)						
Placebo	31.9 ± 20.3	32.9 ± 19.4	37.2 ± 16.2	*p* = 0.729	*p* = 0.965	*p* = 0.796
L-citrulline	35.7 ± 17.5	31.6 ± 14.6	33.5 ± 17.6			
Augmentation index @75 (%)						
Placebo	26.7 ± 20.5	27.6 ± 20.8	38.0 ± 35.4	*p* = 0.767	*p* = 0.249	*p* = 0.969
L-citrulline	30.2 ± 25.4	26.1 ± 14.6	35.0 ± 26.4			
Forward wave pressure (mmHg)						
Placebo	43.3 ± 5.6	31.9 ± 5.1	32.7 ± 4.8	*p* = 0.896	*p* = 0.816	*p* = 0.503
L-citrulline	31.5 ± 4.5	31.6 ± 5.6	32.4 ± 6.1			
Backward wave pressure (mmHg)						
Placebo	17.2 ± 4.0	17.5 ± 4.2	17.8 ± 4.4	*p* = 0.769	*p* = 0.659	*p* = 0.631
L-citrulline	16.8 ± 5.3	16.5 ± 3.2	17.0 ± 4.2			
C-f pulse wave velocity (m/s)						
Placebo	7.3 ± 1.7	7.1 ± 1.4		*p* = 0.251	*p* = 0.251	*p* = 0.666
L-citrulline	7.7 ± 1.8	7.7 ± 1.8				

c-f, carotid–femoral; mmHg, millimeter of mercury; @75, standardized at 75 beats per minute; m/s, meters per second; C × T, two-way interaction between condition and time; C, main effect of condition; T, main effect of time.

## Data Availability

Data from the current study are available from the corresponding author upon reasonable request.

## References

[1] Roth G.A., Mensah G.A., Johnson C.O., Addolorato G., Ammirati E., Baddour L.M., Barengo N.C., Beaton A.Z., Benjamin E.J., Benziger C.P. (2020). Global Burden of Cardiovascular Diseases and Risk Factors, 1990–2019: Update From the GBD 2019 Study. J. Am. Coll. Cardiol..

[2] Kaess B.M., Rong J., Larson M.G., Hamburg N.M., Vita J.A., Levy D., Benjamin E.J., Vasan R.S., Mitchell G.F. (2012). Aortic stiffness, blood pressure progression, and incident hypertension. JAMA.

[3] Mitchell G.F. (2014). Arterial stiffness and hypertension: Chicken or egg?. Hypertension.

[4] Safar M.E., Asmar R., Benetos A., Blacher J., Boutouyrie P., Lacolley P., Laurent S., London G., Pannier B., Protogerou A. (2018). Interaction Between Hypertension and Arterial Stiffness. Hypertension.

[5] Mancia G., Bombelli M., Facchetti R., Madotto F., Corrao G., Trevano F.Q., Grassi G., Sega R. (2007). Long-term prognostic value of blood pressure variability in the general population: Results of the Pressioni Arteriose Monitorate e Loro Associazioni Study. Hypertension.

[6] Willum-Hansen T., Staessen J.A., Torp-Pedersen C., Rasmussen S., Thijs L., Ibsen H., Jeppesen J. (2006). Prognostic value of aortic pulse wave velocity as index of arterial stiffness in the general population. Circulation.

[7] Lundberg J.O., Weitzberg E. (2022). Nitric oxide signaling in health and disease. Cell.

[8] Tousoulis D., Kampoli A.M., Tentolouris C., Papageorgiou N., Stefanadis C. (2012). The role of nitric oxide on endothelial function. Curr. Vasc. Pharmacol..

[9] Stamler J.S., Meissner G. (2001). Physiology of nitric oxide in skeletal muscle. Physiol. Rev..

[10] Chatzinikolaou P.N., Margaritelis N.V., Chatzinikolaou A.N., Paschalis V., Theodorou A.A., Vrabas I.S., Kyparos A., Nikolaidis M.G., Cobley J.N., Davison G.W. (2022). Oxygen Transport. A Redox O2dyssey. Oxidative Eustress in Exercise Physiology.

[11] Margaritelis N.V., Paschalis V., Theodorou A.A., Kyparos A., Nikolaidis M.G. (2018). Antioxidants in Personalized Nutrition and Exercise. Adv. Nutr..

[12] Tejero J., Shiva S., Gladwin M.T. (2019). Sources of Vascular Nitric Oxide and Reactive Oxygen Species and Their Regulation. Physiol. Rev..

[13] Morris S.M. (2004). Enzymes of arginine metabolism. J. Nutr..

[14] Bescos R., Sureda A., Tur J.A., Pons A. (2012). The effect of nitric-oxide-related supplements on human performance. Sports Med..

[15] Kerksick C.M., Wilborn C.D., Roberts M.D., Smith-Ryan A., Kleiner S.M., Jager R., Collins R., Cooke M., Davis J.N., Galvan E. (2018). ISSN exercise & sports nutrition review update: Research & recommendations. J. Int. Soc. Sports Nutr..

[16] He Y., Liu J., Cai H., Zhang J., Yi J., Niu Y., Xi H., Peng X., Guo L. (2021). Effect of inorganic nitrate supplementation on blood pressure in older adults: A systematic review and meta-analysis. Nitric Oxide.

[17] Jackson J.K., Patterson A.J., MacDonald-Wicks L.K., Oldmeadow C., McEvoy M.A. (2018). The role of inorganic nitrate and nitrite in cardiovascular disease risk factors: A systematic review and meta-analysis of human evidence. Nutr. Rev..

[18] Allerton T.D., Proctor D.N., Stephens J.M., Dugas T.R., Spielmann G., Irving B.A. (2018). l-Citrulline Supplementation: Impact on Cardiometabolic Health. Nutrients.

[19] van de Poll M.C., Siroen M.P., van Leeuwen P.A., Soeters P.B., Melis G.C., Boelens P.G., Deutz N.E., Dejong C.H. (2007). Interorgan amino acid exchange in humans: Consequences for arginine and citrulline metabolism. Am. J. Clin. Nutr..

[20] Schwedhelm E., Maas R., Freese R., Jung D., Lukacs Z., Jambrecina A., Spickler W., Schulze F., Boger R.H. (2008). Pharmacokinetic and pharmacodynamic properties of oral L-citrulline and L-arginine: Impact on nitric oxide metabolism. Br. J. Clin. Pharmacol..

[21] Moinard C., Maccario J., Walrand S., Lasserre V., Marc J., Boirie Y., Cynober L. (2016). Arginine behaviour after arginine or citrulline administration in older subjects. Br. J. Nutr..

[22] Windmueller H.G., Spaeth A.E. (1981). Source and fate of circulating citrulline. Am. J. Physiol..

[23] Aguayo E., Martínez-Sánchez A., Fernández-Lobato B., Alacid F. (2021). L-Citrulline: A Non-Essential Amino Acid with Important Roles in Human Health. Appl. Sci..

[24] Maharaj A., Fischer S.M., Dillon K.N., Kang Y., Martinez M.A., Figueroa A. (2022). Effects of L-Citrulline Supplementation on Endothelial Function and Blood Pressure in Hypertensive Postmenopausal Women. Nutrients.

[25] Sanchez-Gonzalez M.A., Koutnik A.P., Ramirez K., Wong A., Figueroa A. (2013). The effects of short term L-citrulline supplementation on wave reflection responses to cold exposure with concurrent isometric exercise. Am. J. Hypertens..

[26] Wong A., Alvarez-Alvarado S., Jaime S.J., Kinsey A.W., Spicer M.T., Madzima T.A., Figueroa A. (2016). Combined whole-body vibration training and l-citrulline supplementation improves pressure wave reflection in obese postmenopausal women. Appl. Physiol. Nutr. Metab..

[27] Wong A., Chernykh O., Figueroa A. (2016). Chronic l-citrulline supplementation improves cardiac sympathovagal balance in obese postmenopausal women: A preliminary report. Auton. Neurosci..

[28] Yang H.H., Li X.L., Zhang W.G., Figueroa A., Chen L.H., Qin L.Q. (2019). Effect of oral L-citrulline on brachial and aortic blood pressure defined by resting status: Evidence from randomized controlled trials. Nutr. Metab..

[29] Chant B., Bakali M., Hinton T., Burchell A.E., Nightingale A.K., Paton J.F.R., Hart E.C. (2018). Antihypertensive Treatment Fails to Control Blood Pressure During Exercise. Hypertension.

[30] Fisher J.P., Young C.N., Fadel P.J. (2015). Autonomic adjustments to exercise in humans. Compr. Physiol..

[31] Jerome L.S., Milliken L.A., Blew R.M., Lohman T., Lohman T., Milliken L. (2020). Body Composition Field Methods. ACSM’s Body Composition Assessment.

[32] Siri W.E. (1993). Body composition from fluid spaces and density: Analysis of methods. 1961. Nutrition.

[33] Butlin M., Qasem A. (2017). Large Artery Stiffness Assessment Using SphygmoCor Technology. Pulse.

[34] Andrews A.W., Thomas M.W., Bohannon R.W. (1996). Normative values for isometric muscle force measurements obtained with hand-held dynamometers. Phys. Ther..

[35] Theofilou G., Ladakis I., Mavroidi C., Kilintzis V., Mirachtsis T., Chouvarda I., Kouidi E. (2022). The Effects of a Visual Stimuli Training Program on Reaction Time, Cognitive Function, and Fitness in Young Soccer Players. Sensors.

[36] Baltzopoulos V., Gleeson N.P., Eston R., Reilly T. (2001). Skeletal muscle function. Kinanthropometry and Exercise Physiology Laboratory Manual: Tests, Procedures and Data.

[37] Faul F., Erdfelder E., Lang A., Buchner A. (2007). G*Power 3: A flexible statistical power analysis program for the social, behavioral, and biomedical sciences. Behav. Res. Methods.

[38] Chrysant S.G., Chrysant G.S. (2014). The age-related hemodynamic changes of blood pressure and their impact on the incidence of cardiovascular disease and stroke: New evidence. J. Clin. Hypertens..

[39] Pinto E. (2007). Blood pressure and ageing. Postgrad. Med. J..

[40] Ochiai M., Hayashi T., Morita M., Ina K., Maeda M., Watanabe F., Morishita K. (2012). Short-term effects of L-citrulline supplementation on arterial stiffness in middle-aged men. Int. J. Cardiol..

[41] Theodorou A.A., Chatzinikolaou P.N., Margaritelis N.V., Christodoulou F., Tsatalas T., Paschalis V. (2023). Short-Term L-Citrulline Supplementation Does Not Affect Inspiratory Muscle Oxygenation and Respiratory Performance in Older Adults. Nutrients.

[42] Theodorou A.A., Zinelis P.T., Malliou V.J., Chatzinikolaou P.N., Margaritelis N.V., Mandalidis D., Geladas N.D., Paschalis V. (2021). Acute L-Citrulline Supplementation Increases Nitric Oxide Bioavailability but Not Inspiratory Muscle Oxygenation and Respiratory Performance. Nutrients.

[43] Khalaf D., Kruger M., Wehland M., Infanger M., Grimm D. (2019). The Effects of Oral l-Arginine and l-Citrulline Supplementation on Blood Pressure. Nutrients.

[44] Figueroa A., Alvarez-Alvarado S., Ormsbee M.J., Madzima T.A., Campbell J.C., Wong A. (2015). Impact of L-citrulline supplementation and whole-body vibration training on arterial stiffness and leg muscle function in obese postmenopausal women with high blood pressure. Exp. Gerontol..

[45] Figueroa A., Trivino J.A., Sanchez-Gonzalez M.A., Vicil F. (2010). Oral L-citrulline supplementation attenuates blood pressure response to cold pressor test in young men. Am. J. Hypertens..

[46] Jaime S.J., Nagel J., Maharaj A., Fischer S.M., Schwab E., Martinson C., Radtke K., Mikat R.P., Figueroa A. (2022). L-Citrulline supplementation attenuates aortic pulse pressure and wave reflection responses to cold stress in older adults. Exp. Gerontol..

[47] Ashley J., Kim Y., Gonzales J.U. (2018). Impact of l-citrulline supplementation on oxygen uptake kinetics during walking. Appl. Physiol. Nutr. Metab..

[48] Morimoto S., Ichihara A. (2023). Late age at menopause positively associated with obesity-mediated hypertension. Hypertens. Res..

[49] Figueroa A., Alvarez-Alvarado S., Jaime S.J., Kalfon R. (2016). l-Citrulline supplementation attenuates blood pressure, wave reflection and arterial stiffness responses to metaboreflex and cold stress in overweight men. Br. J. Nutr..

[50] Rhee E.J. (2022). The Influence of Obesity and Metabolic Health on Vascular Health. Endocrinol. Metab..

[51] Lewis G.D., Gona P., Larson M.G., Plehn J.F., Benjamin E.J., O’Donnell C.J., Levy D., Vasan R.S., Wang T.J. (2008). Exercise blood pressure and the risk of incident cardiovascular disease (from the Framingham Heart Study). Am. J. Cardiol..

[52] Gonzales J.U., Raymond A., Ashley J., Kim Y. (2017). Does l-citrulline supplementation improve exercise blood flow in older adults?. Exp. Physiol..

[53] Yamashina A., Tomiyama H., Arai T., Hirose K., Koji Y., Hirayama Y., Yamamoto Y., Hori S. (2003). Brachial-ankle pulse wave velocity as a marker of atherosclerotic vascular damage and cardiovascular risk. Hypertens. Res..

[54] Figueroa A., Wong A., Jaime S.J., Gonzales J.U. (2017). Influence of L-citrulline and watermelon supplementation on vascular function and exercise performance. Curr. Opin. Clin. Nutr. Metab. Care.

[55] Edwards D.G., Mastin C.R., Kenefick R.W. (2008). Wave reflection and central aortic pressure are increased in response to static and dynamic muscle contraction at comparable workloads. J. Appl. Physiol..

[56] Stock J.M., Chouramanis N.V., Chirinos J.A., Edwards D.G. (2020). Dynamic and isometric handgrip exercise increases wave reflection in healthy young adults. J. Appl. Physiol..

[57] Connelly P.J., Currie G., Delles C. (2022). Sex Differences in the Prevalence, Outcomes and Management of Hypertension. Curr. Hypertens. Rep..

[58] Reckelhoff J.F. (2023). Mechanisms of sex and gender differences in hypertension. J. Hum. Hypertens..

[59] Stanhewicz A.E., Wenner M.M., Stachenfeld N.S. (2018). Sex differences in endothelial function important to vascular health and overall cardiovascular disease risk across the lifespan. Am. J. Physiol. Heart Circ. Physiol..

[60] Figueroa A., Maharaj A., Kang Y., Dillon K.N., Martinez M.A., Morita M., Nogimura D., Fischer S.M. (2023). Combined Citrulline and Glutathione Supplementation Improves Endothelial Function and Blood Pressure Reactivity in Postmenopausal Women. Nutrients.

